# Return to play after three ipsilateral anterior cruciate ligament reconstructions in an elite soccer player: A case report

**DOI:** 10.1016/j.ijscr.2020.02.027

**Published:** 2020-02-19

**Authors:** José Carlos Noronha, João Pedro Oliveira, João Brito

**Affiliations:** a Portugal Football School, Portuguese Football Federation, Portugal; b Trindade Hospital, Porto, Portugal; c Coimbra Hospital and University Center, Orthopaedic Department, Portugal

**Keywords:** ACL, anterior cruciate ligament, ACL reconstruction, Allograft, Graft failure, Re-revision surgery

## Abstract

**Introduction:**

Despite the reasonable success of ACL reconstruction, some athletes are not able to regain the level of play they once had.

**Presentation of case:**

Here, we report the case of a 32-year-old male professional soccer player who sustained an ACL injury in his right knee. The patient had a history of two prior ipsilateral ACL injuries, which was reconstructed with ipsilateral hamstring autograft (first surgery) and ipsilateral patellar tendon autograft (revision surgery). Imaging examination revealed a small narrowing of the medial femoro-tibial compartment, a complete ACL rupture, partial medial meniscectomy, small cartilage lesions in the medial condyle, a 7° varus knee, an enlarged tibial tunnel, and a femoral tunnel positioned high above the intercondylar roof. A one-step re-revision surgery using a fresh-frozen, cadaveric, non-irradiated Achilles tendon allograft was planned. After surgery, physiotherapy was conducted once per day during 4 months. The patient started running at the 6^th^ month, and returned to full training 8 months after surgery. The player returned to full competitive play 9 months after surgery and has been competing for the last 36 months at the highest level of play without any limitation, inflammation, pain, or perception of instability.

**Conclusion:**

In professional sports, when re-revision ACL reconstruction is indicated and the patient expects to return to competition, surgery should not be delayed. In these cases, the usefulness of Achilles tendon allograft should be taken into consideration for re-revision ACL reconstruction.

## Highlights

• Re-revision ACL reconstruction is recommended to restore joint stability and improve functional outcomes.
• The paucity of information regarding re-revision ACL reconstruction limits prognostics on the ability to return to play after a third ACL rupture.
• In professional sports, when re-revision ACL reconstruction is indicated and the patient expects to return to play, surgery should not be delayed.

## 1 Introduction

Primary anterior cruciate ligament (ACL) reconstruction is a successful procedure, with good to excellent clinical outcomes in 80–90% of patients [1]. In professional football, the majority of players can return to play after ACL reconstruction, but only two-thirds of the injured players can compete at the highest level of play 3 years after surgery [2]. While the number of contralateral ACL ruptures is low, the risk for ipsilateral graft failure is greatest in the first 2 years following ACL reconstruction [2].

In the general population, the rates of a third ACL injury range from 4 to 13% [3,4]. Re-revision ACL reconstruction is recommended to restore joint stability and improve functional outcomes [5]. However, the paucity of information regarding re-revision ACL reconstruction limits any prognostics on a player’s ability to return to pre-injury level of play after a third ACL rupture.

Here, we report the case of a 32-year-old male professional soccer player who returned to play at the elite level following re-revision ACL reconstruction. The current case report follows the Surgical Case Report (SCARE) guidelines [6].

## 2 Presentation of case

A 32-year-old male professional football player presented with as a direct result of the sport. A clinical diagnosis of ACL injury was made. The patient had a history of two prior ipsilateral injuries to the ACL. The first injury occurred when the player was 25 years old. Ipsilateral hamstring autograft (semitendinosus and gracilis tendon) reconstruction was used for the first ACL reconstruction. Return to play occurred 6 months after surgery. The second ACL injury occurred when the player was 28 years old. Ipsilateral bone – patellar tendon – bone autograft was used for the second ACL reconstruction. Return to play occurred 6 months after surgery.

Given the patient’s surgical history, a detailed anatomical study was conducted prior to any further surgical intervention. X-ray showed small narrowing of the medial femoro-tibial compartment, with knee varus of 7°. Magnetic resonance imaging showed complete ACL rupture and partial medial meniscectomy. Also, small cartilage lesions in the medial femoral condyle were detected. Computerized axial tomography showed an excessive enlargement of the tibial tunnel. Three-dimensional pre-operative anatomical medical imaging investigation (PeekMed, Braga, Portugal) established maximal osteolysis in the tibial tunnel of 14.2 mm in the coronal plane and 14.5 mm sagittal plane. Also, computerized axial tomography showed the femoral tunnel was positioned too high in the intercondylar roof, preventing an appropriate orientation and fixation of a new graft. The two previous femoral tunnels had been performed using an antero-medial portal. Therefore, a new outside-in femoral tunnel was planned pre-operatively, keeping the orientation of the previous tibial tunnel.

**Fig. 1:**
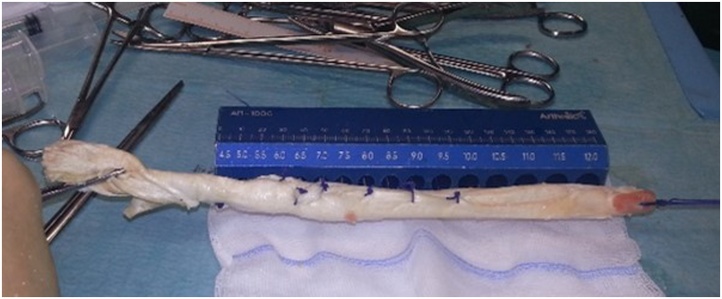
Frozen cadaveric non-irradiated Achilles tendon allograft with calcaneus bone.

Clinically, the patient presented femoro-patellar pain in the contralateral knee, which precluded using a contralateral bone–patellar tendon–bone autograft. Therefore, the patient agreed on using a cadaveric Achilles tendon allograft for the re-revision ACL reconstruction. For this purpose, a cadaveric non-irradiated Achilles tendon allograft with calcaneus bone was selected (Fig. 1). Since ipsilateral osteolysis of the tibial tunnel has been detected, a one-step re-revision surgery was accorded with the patient. This allowed a one-step reconstruction with no donor site morbidity. The calcaneus bone portion of the allograft was selected to fill the tibial bone defect.

During surgery, any pneumatic tourniquet was used. An outside-in femoral tunnel technique was employed with the knee at 90° of flexion. This permitted to easily avoid the previous inappropriate femoral tunnels and allowed a more confident proximal graft fixation. Contrary to the common procedure of keeping the bone block in the femoral tunnel, the allograft was inserted through the joint from the tibial tunnel (distal to proximal) keeping the bone block in the tibial defect. Resorbable screws were used for graft fixation in both tunnels. Graft fixation has been recommended to occur with the knee flexed to 30°. However, the strong cadaveric Achilles tendon allograft was over-tensioned, and definitive graft fixation occurred with the knee in full extension.

**Fig. 2:**
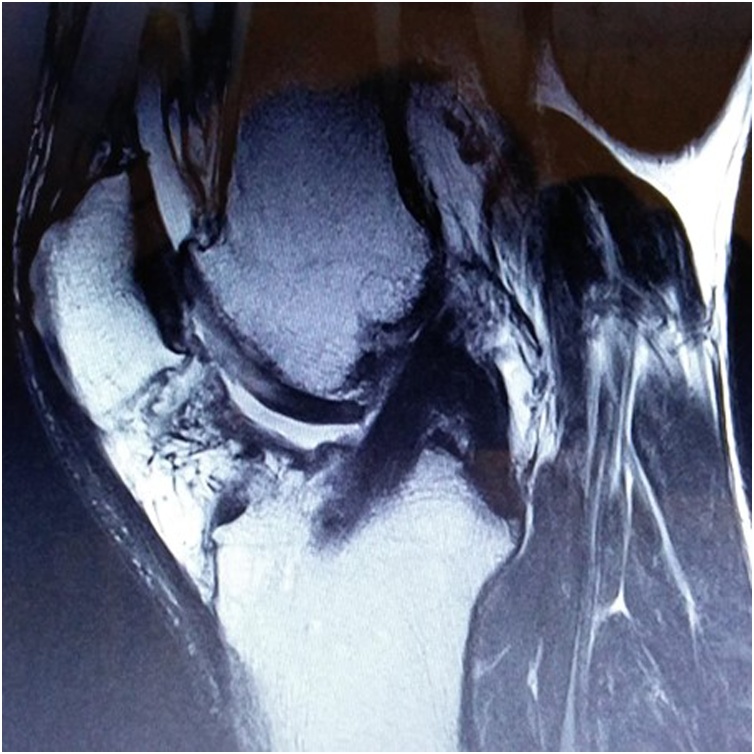
Magnetic resonance imaging conducted 4 months after surgery, showing normal graft thickness and T2-hypointense homogeneous signal, regular borders, and full graft integration in the tunnels.

Post-surgery, the patient used crunches for 4 weeks, with tolerable body weight during this period. Full range of motion of the knee was allowed. During this 4-week period, slight muscle atrophy was detected. Physiotherapy rehabilitation was conducted once per day during 4 months. Magnetic resonance imaging was conducted 4 months after surgery, showing normal graft thickness and T2-hypointense homogeneous signal, regular borders, and full graft integration in the tunnels (Fig. 2). The patient started running at the 6^th^ month, and returned to full training without restrictions 8 months after surgery. Return to competitive match play occurred 9 months after surgery. The player is still competing at the elite professional level without any limitation, inflammation, pain or perception of instability over the last 36 months. Clinically, the patient presents normal joint mobility, negative pivot shift and normal Lachman test outcome.

## 3 Discussion

To the best of our knowledge, this is the first clinical report of re-revision ACL reconstruction in a professional elite athlete. Re-revision ACL reconstruction is technically challenging, mostly due to tunnel placement, bone stock, tunnels osteolysis, limited graft availability, complexity in acquiring stable graft fixation and concomitant lesions (e.g. ligamentar, meniscal and osteochondral). It is noteworthy, however, the paucity of literature available on re-revision ACL reconstruction. In fact, a recent systematic review on the outcomes and risk factors for re-revision ACL reconstruction showed that only 6 articles could be considered for analysis [5].

Indication for ACL re-revision surgery essentially depends on the patient’s sport and concomitant professional commitments. Perceived instability in daily living activities is a strong argument for conduction a re-revision, but age, sports level, motivation, and career perspectives should all be taken into account before any re-revision ACL reconstruction is conducted. We believe that ACL re-revision surgery should not be considered a salvage procedure. Contrary, the whole process should be carefully and individually addressed. The patient should be noticed that failure rates in ACL re-revision are 8–13% higher than those reported for primary ACL reconstruction, and return to prior athletic activity level ranges from 20 to 80% [7,8]. Decreased Tegner activity scale scores should be expected at final follow-up (range: 2.6–5 years postoperative) when compared with pre-failure or after first-time ACL revision reconstruction. In the case presented here, despite being an elite football player, Tegner activity scale scores were maintained at level 10 even 9 months after surgery.

Younger age (<20 years) and the use of allograft tissue at the time of ACL revision are considered independent risk factors of subsequent surgery on the ipsilateral knee [9]. However, perpetuation of knee instability episodes increases the risk for meniscal and chondral injuries. Though, in these cases, we strongly believe that re-revision ACL reconstruction should not be delayed.

Surgical revision of a failed ACL reconstruction requires thorough preoperative planning. First, the factors that might have caused graft failure should be deeply clarified. The tibial slope and other intrinsic factors (e.g. anatomic features and ligamentous structures) should be evaluated prior to surgery, especially in cases of non-traumatic graft failure. We planned this surgery using pre-operative three-dimensional reconstruction imaging software, in order to establish the dimension and orientation of the tunnels, as well as guarantee appropriate graft fixation. Though, some surgical technical pearls have been used. Given the inappropriate previous femoral tunnels, a new femoral tunnel was drilled in an outside-in fashion with the knee at 90°. The non-irradiated cadaveric Achilles tendon allograft with good bone block was appropriate given the limited autograft availability in the current patient. The common surgical procedure is to keep the bone block in the femoral tunnel. However, a different procedure was used here: the bone block was used to fill the osteolysis detected around the tibial tunnels.

In our opinion, in professional sports, when re-revision ACL reconstruction is indicated and the patient expects to return to competition, surgery should not be delayed. The worst-case scenario is chondral and meniscal injuries, which will decrease the final long-term outcomes. Though, in older professional athletes, the usefulness of Achilles tendon allograft should always be taken into consideration for re-revision ACL reconstruction. Still, large multicentre cohorts are key in order to properly establish the effective rates, risk factors, reconstruction strategies and final outcomes of multiple ACL reconstructions.

## 4 Conclusion

Here, we report the case of a professional soccer player who sustained an third ACL injury in his right knee. The patient had a history of two prior ipsilateral ACL injuries. For the re-revision surgery, we used a fresh-frozen, cadaveric, non-irradiated Achilles tendon allograft. The player returned to full competitive play 9 months after surgery and has been competing for the last 36 months at the highest level of play without any limitation, inflammation, pain, or perception of instability.

## Funding

This research did not receive any specific grant from funding agencies in the public, commercial, or not-for-profit sectors.

## Ethical approval

Ethical approval was not required and patient-identifying knowledge was not presented in the report.

## Consent

Written informed consent has been obtained from the patient for the publication of this case report and any accompanying images.

## Author contribution

JCN and JPO participated in treatment of the patient, collected case details, literature search and drafted the manuscript. JB collected case details and literature search, and drafted the manuscript. All authors read and approved the final manuscript.

## Registration of research studies

Not applicable.

## Guarantor

José Carlos Noronha.

## Provenance and peer review

Not commissioned, externally peer-reviewed.

## Declaration of Competing Interest

The authors have no conflicts of interest.
